# Assessment of patient safety culture: what tools for medical students?

**DOI:** 10.1186/s12909-016-0778-y

**Published:** 2016-09-29

**Authors:** M. Chaneliere, F. Jacquet, P. Occelli, S. Touzet, V. Siranyan, C. Colin

**Affiliations:** 1 Health Services and Performance Research (HESPER), 162 Avenue Lacassagne Bât A—F69424, Lyon, cedex 03 France; 2 Claude Bernard University Lyon 1 (UCBL), 8 avenue Rockefeller, F 69373, Lyon, Cedex 08 France; 3 Hospices Civils de Lyon, Pôle IMER, 162 Avenue Lacassagne Bât A—F69424, Lyon, cedex 03 France; 4 Department of Family Practice—UCB Lyon, 1 - 8 av Rockefeller 69373, Lyon, cedex 08 France

**Keywords:** Patient safety culture, Medical student, Assessment, Survey

## Abstract

**Background:**

The assessment of patient safety culture refers mainly to surveys exploring the perceptions of health professionals in hospitals. These surveys have less relevance when considering the assessment of the patient safety culture of medical students, especially at university or medical school. They are indeed not fully integrated in care units and constitute a heterogeneous population. This work aimed to find appropriate assessment tools of the patient safety culture of medical students.

**Methods:**

Systematic review of the literature. Surveys related to a care unit were excluded. A typology of the patient safety culture of medical students was built from the included surveys.

**Results:**

Eighteen surveys were included. In our typology of patient safety culture of medical students (15 dimensions), the number of dimensions explored by survey (n) ranged from 1 to 12, with 6 “specialized” tools (*n* ≤ 4) and 12 “global” tools (*N* ≥ 5). These surveys have explored: knowledge about patient safety, acknowledgment of the inevitability of human error, the lack of skills as the main source of errors, the errors reporting systems, disclosure of medical errors to others health professionals or patients, teamwork and patient involvement to improve safety in care.

**Conclusions:**

We recommend using Wetzel’s survey for making an overall assessment of the patient safety culture of medical students at university. In a specific purpose—e.g. to assess an educational program on medical error disclosure—the authors recommend to determine which dimensions of patient safety will be taught, to select the best assessment tool. Learning on patient safety should however be considered beyond the university. International translations of tools are required to create databases allowing comparative studies.

**Electronic supplementary material:**

The online version of this article (doi:10.1186/s12909-016-0778-y) contains supplementary material, which is available to authorized users.

## Background

For many years adverse events (AE) have been reported in the literature under various names [1] e.g. Patient Safety Incident (PSI). The book “To err is human” [2], led to international awareness of their frequency and gravity. Patient safety (PS) should be a constant concern of all healthcare professionals (HP) who should all learn acceptable patient safety culture (PSC) during their initial training. The International Classification for Patient Safety (ICPS) [3] described “patient safety” as “the reduction of risk of unnecessary harm associated with healthcare to an acceptable minimum”. This is a definition integrated in a systemic approach as described by James Reason several years ago [4]. The concept of “safety culture” was first used by the International Atomic Energy Agency after the Chernobyl accident [5] and then in healthcare with PSC. Sammer et al [6] have identified seven factors affecting PSC (leadership, teamwork, evidence-based medicine, communication, learning, “just culture”, and patient-centered) but several definitions of PSC exist [7–9]. The European Society for Quality in Health Care [7] defined it as “an integrated pattern of individual and organizational behaviour, based upon shared beliefs and values, that continuously seeks to minimize patient harm which may result from the processes of care delivery”. It constitutes a functional and opened definition allowing an assessment of the patient safety culture in different groups of health professionals, according to several dimensions e.g. “use of a systemic analysis approach, “PSI reporting”, “disclosure of adverse event to patient” etc. Several self-administered surveys [10] allow graduate d professionals to assess their PSC e.g. in health units the “Hospital Survey On Patient Safety Culture” (HSOPSC) is frequently used [11]. Considering trainings dedicated to patient safety performed at the University of Lyon 1, the authors asked themselves what tools should be use to assess patient safety culture of medical students. The purpose of our work was to identify appropriate tools to assess patient safety culture for the medical students (PSMS).

## Methods

### First step: literature search

A systematic review of the literature was conducted. Research questioned the “Medline” database, “The Cochrane Library” and “Web of Science”. Tables 1 and 2 present the keywords and search strategies. The keywords related to the assessment process fit in the four levels described by Kirkpatrick [12] for the assessment of training programs, i.e. Knowledge, Skills, Abilities and Behaviours, sometimes called “KSAB” axes. The research focused on articles published between 2000 and 2013. An additional manual search was performed on the sites of several international and national agencies involved in care safety, including the Agency for Healthcare Research and Quality, the National Patient Safety Agency and the WHO. Table 3 summarizes the criteria for inclusion and exclusion. Two physicians with expertise in the field of care safety (MC and FJ) selected the articles jointly during working sessions. They ensured strict application of the inclusion and exclusion criteria, first to the title and abstract of each article. The articles selected after this step were read fully before final inclusion.

**Table 1:** Keywords

Fields	Keywords
Population	medical student* medical trainee* intern*, resident*, house staff
Overall dimension	patient safety culture
Dimension related to the medical error	medical error* medical mistake*
Cognitive field (“What students know”)	knowledge*
Competence field (“What students can do”)	skill*
Psychological field (“What students think/feel do”)	attitude* belief* value*
Behaviour field (“What students are doing”)	behaviour*

* has been added to singular to identify their plural forms in some databases

**Table 2:** Search Strategy

Number	Strategy
1	“patient safety culture” AND (“medical student*” OR “intern*” OR “resident*” OR “medical trainee*” OR “house staff”)
2	(“attitude*” OR “knowledge*” OR “value*” OR “belief*” OR “behaviour*”OR “skill*”) AND (“patient safety”) AND (“medical student*” OR “intern*” OR “resident*” OR “medical trainee*” OR “house staff”)
3	(“attitude*” OR “ knowledge*” OR “value*” OR “belief*” OR “behaviour*” OR “skill*”) AND (“medical error*” OR “medical mistake*”) AND (“medical student*” OR “intern*” OR “resident*” OR “medical trainee*” OR “house staff”

* has been added to singular to identify their plural forms in some databases

**Table 3:** Criteria for inclusion and exclusion

Inclusion Criteria	
• Survey fully published in article	
Population	• Medical students only • From the first year until graduation year • Any medical specialty
Explored fields (*n* ≥ 1)	• Psychological o Attitudes of medical students o Perceptions of the dimensions of patient safety • Behaviour o Students’ actions promoting patient safety • Knowledge and skills in patient safety
• Survey in English	
Exclusion Criteria	
• Survey restricted to a simple assessment of the psycho-emotional impact of a Patient safety incident on student	
• Survey related to a hospital care unit	
• Survey related to an ambulatory care unit	

An analysis of the surveys was conducted following the same methodology as Singla et al [10]. It requires to build a specific typology of the dimensions of PSMS by looking into characteristics and items of every surveys (second step). Then, the typology must be used for analysing each questionnaire (third step).

### Second step: building of a typology of PSMS and dimensions explored by the surveys

A content analysis was conducted to find all items of the surveys exploring the PSC. These items were grouped together in distinct sub-dimensions and then in main dimensions (MC and FJ). The experts reached a consensus in case of divergence regarding the choice of a sub-dimension or a dimension. A sub-dimension was defined as “the ability to explore a specific aspect or an area of the PSC, unexplored by any other sub-dimension”. Some sub-dimensions were obtained by reading questionnaires because the items were often grouped into areas of PSC that they are intended to measure. Others sub-dimensions were obtained by gathering items related with a same aspect of the PSC. When all sub-dimensions were identified, the experts gathered them in main dimensions, exploring a same global aspect of the PSC. Choices of dimensions (number and type) were confirmed by the other authors, engaged in quality improvement and patient safety.

### Third step: analysis of surveys

Each included survey was finally analysed using the typology previously built (number of items per dimension of the typology) and using some other judgment criteria: country and year of completion, the context in which the study was performed, the population (description and total population), the answering procedures, and the existence of a statistical validation or metrological control of the survey. These criteria were chosen according to their use in other works [10] and their availability inside tools.

## Results

### First step: identification of surveys

**Fig. 1:**
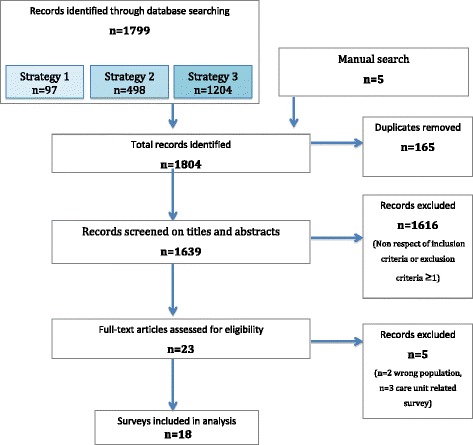
Flow-Chart

Research in databases identified 1799 publications; 5 articles were from manual searches; 165 were duplicates; 1616 items did not fit the inclusion criteria or had at least one exclusion criterion; finally, 18 [13–30] surveys were included (Fig. 1).

### Second step: typology of PSMS and explored dimensions by surveys

The 18 surveys had a total of 423 items. The median number of items was 23 (from 8 [19] to 48 [23]). After analysing and grouping all the items in sub-dimensions (*n* = 48) then in main dimensions, a typology of the patient safety culture of medical students in 15 dimensions was chosen. The Table 4 shows: the PSMS typology, the number of dimensions of PSMS explored per survey and the number of surveys per dimension of the typology. The six most frequently explored dimensions are: reporting an error or a PSI to his/her superiors (*n* = 14 surveys), MS attitudes regarding the disclosure of medical errors (ME) to patients (*n* = 13), use of a PSI reporting system (*n* = 13), analysis of PSI and ME (*n* = 11), knowledge about patient safety (*n* = 11) and, finally, systemic and individual responsibilities (*n* = 10). In contrast, the three dimensions less frequently explored are knowledge about care protocols and procedures (*n* = 2), consciousness of its own limits (*n* = 3) and patient’s involvement in care safety (*n* = 3).

**Table 4:** Number of items per dimension and per survey

DIMENSIONS		SURVEYS																		SURVEYS PER DIMENSION (number)
		Coyle	Sorokin	Madigosky	Moskowitz	Kaldjian	Muller	Kerfoot	Patey	Bechtold	Carruthers	Flin	Logio	Leung.1	Friedman	Leung.2	Dudas	White	Wetzel	
TYPOLOGY OF THE PATIENT SAFETY CULTURE OF MEDICAL STUDENTS	Knowledge about patient safety			5	2		4	15	7	1	1	9		7		5			1	11
	Medical errors occurring in care			1							3	1		1		1	2		3	7
	Systemic and individual responsibilities		3	3	2		4			5	4			3		4		2	4	10
	Analysis of PSI (Patient Safety Incident)		1	3	1				9	5	1	7		3		2	1		1	11
	Notification of PSI (reporting system)	13	2	4	2				2	3	2	1	4	1	1	1			3	13
	Announcement of an error to peers or one’s senior	1		5		3	2		14	1	2	8	4	1	13	1	2		12	14
	Disclosure of a medical error to patients		2	2	1	19	3		1	2	1			2	1	3		8	1	13
	Student witness of an error or PSI			1	3				8		1	1		1		1	2		5	9
	Student knows and reports its limits		1		1										5					3
	Teamwork		3								2						2		2	4
	Managing the workload		2		2						3								3	4
	Protocols and care procedures		2		5															2
	School teaching dedicated to patient safety			2					1		3			2		3			3	6
	Patient involvement in their own safety		1								2								2	3
	Psycho-emotional impact of an error on the doctor			1		1	4		5	1		4		1				1		8
Unclassifiable items			2	4	4		6		1	2	1	2		3	3	2	6		3	
Total number of items per survey		14	19	31	23	23	23	15	48	20	26	33	8	25	23	23	15	11	43	
DIMENSIONS PER SURVEY (number)		2	9	10	9	3	5	1	8	7	12	7	2	10	4	9	5	3	12	

### Third step: Description of surveys

A table which summarizes the characteristics of each survey is included in Additional file 1. There are links between several tools: Flin’s [20] are based on the Patey questionnaire [23]; Carruthers’ [21] are based on the Sorokin questionnaire [29]; the two questionnaires of GK Leung [16, 18] are derived from the Madigosky questionnaire [28] and AP Wetzel [13] upgraded the Carruthers questionnaire [21].

Ten surveys were designed as part of an overall evaluation of the PSC conducted at the university (excluding specific intervention), seven were designed to assess the impact of a teaching program dedicated to MS. Only one was designed to create a specific research tool for assessing PSMS (original research).

Several questionnaires address to various years of medical school: from the first or second year of cursus [20, 28], the third or the fourth year [16, 18, 26, 27] and the last years of cursus as residents or fellowships [19, 22, 23, 30]. Some questionnaires address to every years of medical school without any distinction [13, 15, 21, 24, 25].

The tools were classified into two categories according to the number of dimensions of the typology of the PSMS they explore: “specific” surveys (*n* = 6) targeting up to four-dimensions and “general” surveys (*n* = 12) exploring five dimensions or more. The “specific” tools mainly explore the knowledge of PSI or errors and the use of reporting systems. They are often conducted after dedicated teaching programs. The “general” surveys explore dimensions of PSI reporting, announcement or disclosure, systemic / personal liability and PSI analysis by MS. Items follow each other without any special organization in the forms except those of Carruthers [21] and Wetzel [13], in which there are clusters around the main axes or types of dimension (e.g. PSI reporting or disclosure of ME). Answers are given anonymously, in paper or electronic form. The answers are often scored on Likert scales with 5 (or 7) degrees. Multiple-choice questions are used less frequently, with questions closed at two modalities, “yes/no”, or free responses. There is no preferred mode according to the dimension. The surveys allow assessing the PSC of a group of students after aggregating all their responses. Of the five surveys with statistical evaluations, only two provided a more robust metrological evaluation [13, 21]. Only two tools [13, 21] could be used to rate a score for several dimensions of the patient safety culture, after assessing a medical student. In the other tools, the items are not gathered by dimensions but only by aspect of the PSC.

## Discussion

### Different uses

This literature review was conducted to identify appropriate tools dedicated to the assessment of PSMS. Results suggest that the answer would vary depending on the wished use: global or specific assessment.

When considering a global assessment of the PSC, Wetzel’s tool [13] seems to provide a suitable tool in terms of assessing overall perceptions of medical students on safety as part of their initial training. This tool addresses essential dimensions [9–11] such as “knowledge about patient safety”, “acknowledgment of the inevitability of human error”, “attitudes toward lack of skill as a main source of errors”, “PSI reporting system”, “disclosure of medical errors to others HP or patients”, “teamwork” and “the patient’s involvement in their own safety”. However, it does not evaluate the aspects related to “process and protocols”, which are more related to a care unit and can be explored through other tools [10, 11]. The Wetzel’s tool provides individual scores of dimensions of PSC. This allows a mapping of the PSC of a student among others MS what is suitable to suggest specific training actions.

Kerfoot’s tool [24] was the only questionnaire designed to assess students’ knowledge about patient safety. However, its wider use (i.e. international) would require a real adaptation (e.g. national epidemiological data for every country). White’s survey [14] explored attitudes regards disclosure of ME to patients. The main steps recommended are explored: description of the fact, explicit formulation that an error has occurred, apologies and description of actions to improve patient safety. This tools also investigates if a serious error would have an impact on the content of the disclosure (responses comparing two groups of MS who received a different vignette). Finally, it assesses the acknowledgment by students of their responsibility (legal issues related to PSI and decrease of patient’s confidence level). For example, this tool could be used for assessment of MS inside programs using experimental learning (role play and simulations).

Seven surveys were specifically designed to assess the impact of a health care safety education program for MS. This explains a higher frequency of the assessment of the dimension “knowledge about patient safety” in relation to hospital surveys mentioned in the work of Sammer [6].

Medical students work under the supervision of physician faculty members or clinical tutors, whether in a hospital or community setting. The clinical supervision regarding patient safety is often analysed through the process of “error reporting”. This is a significant element. These considerations reduce the range of these tools: they should only be used in initial training (e.g. assessing the impact of a program on the development of safety culture).

### Reliability and adaptation

Every questionnaire should undergo evaluation of its psychometric properties before it is relied on for making any surveys. Several elements can affect psychometric properties of a questionnaire: number of items, response options, recall period, time between administrations, respondent burden, and scoring algorithm etc. However, three characteristics should be explored: measurements should be reliable and reproducible, right elements should be measured in the tool and even a minimal change could be explored. The evaluation of the psychometric properties of the Wetzel’s tool [13] sounds reasonably robust regarding these core characteristics.

Considering an international use of these tools, it would be necessary to translate them. Several guidelines on methods for the translation of a questionnaire exist [31, 32] but all have the same process: first a translation by an Expert Committee, then a work to identify any cultural differences and finally a translation back. The translation must also respect the way the scales measure dimensions: an “agreement in attitude” (“what MS thinks do”) differs from a “frequency of behaviour” (“What MS do”). According to us, a pilot study on a test sample with focus groups or interviews should be interested (work with a department of linguistics).

### Patient Safety Culture and medical student

The studies leading to a medical degree are specific to a country (organization, cost, number of years etc.). However, the MeSH vocabulary includes “all individuals enrolled in a school of medicine or a formal educational program in medicine” under “Medical Students”. The assessment of PSMS can refer to the assessment of knowledge, skills, attitudes and behaviours [12] acquired by MS during their studies. Segmenting the notion of PSC between graduate professionals and professionals in initial training remains questionable. The definition of the European Society for Quality of Care [7] was chosen because it is not attached to a care organization, nor does it determine professionals according to their level of learning. The term “patient safety culture” is also discussed and some prefer the term “patient safety climate”. The literature does not provide a definite answer probably because the two concepts are closely related (safety climate consists of the surface elements of the safety culture [33]). Our research strategy aimed to avoid problems related to these choices. Hafferty [34] has described three types of training methods: formal trainings (formal programs taught at the Faculty), informal trainings (not pre-determined and interpersonal student—teacher) and “hidden teaching” or “hidden curriculum” (unseen, corresponding to a set of organizational and cultural influences). At the university or at the medical school, the assessment of PSMS should be done through a survey independent of care units. Indeed, the knowledge and skills of MS vary depending on their level of learning or their involvement in specific trainings. Furthermore, they are not fully integrated in one care unit even after a whole semester of internship. For students, the notion of unity is more related to a common core of academic training and less to group work in a care unit. The years of medical school preceding graduate medical education are split into a preclinical phase and a clinical phase (dedicated to learning in the clinical setting), but MS frequently have clinical experiences before the clinical phase (according to countries, learnings before medical school etc.) In addition, learnings dedicated about patient safety can concern medical students from various years of the curriculum. So when choosing a tool, it seems preferable to focus on skills or elements explored by each survey rather than the year of medical curriculum theoretically addressed by the survey. Thus the attitudes of the senior doctor during the clerkship e.g. when a student reported him an error, has also a strong influence on his PSC [35, 36]. For that reason, some authors [37] recommend that trainings on patient safety should always follow a dual approach with a formal side in the faculties or medical schools, applied and reinforced during practice in care units [38].

### Typology and dimensions of PSMS

Due to the lack of a specific typology of PSMS in the literature, we adopted the same methodology used by Singla [10] in their review of hospital assessment tools. Questionnaires without any assessment of their psychometric properties were included. That constitutes perhaps a limit of the method, but this approach allowed to reach a consequent number of items to identify dimensions of the PSMS and to build the typology. No mathematical weighting was applied on the 423 items because there is no consensus in the literature on the relevance of the different dimensions of PSMS, or on the links established between dimensions. Our 15-dimension model has allowed a detailed typology without redundancy. The dimensions of PSMS identified in our study are closed to those identified by Singla [10].

Authors thought about the clearest way to expose safety processes explored in the surveys and tried to link each dimension of the typology with the seven factors affecting PSC identified by Sammer CE et al [6]. But some dimensions (e.g. “protocols and care procedures”) could be indexed under several factors (e.g. “Communication”, “Learnings”, “Patient-Centered” and “Teamwork”) so the results were unclear. On the other side, the gathering of some dimensions of our typology may be considered to obtain a smaller model with only 12 dimensions by merging the dimensions “to disclose a medical error to patient” with “to disclose a medical error to one’s peers”; “reporting one’s limits” with “management workload”; “teaching dedicated to safety” with “knowledge about patient safety”.

## Conclusion

Wetzel’s questionnaire [13] seems to provide an appropriate tool in terms of assessing overall perceptions of medical students on safety as part of their initial training. However, it still requires some supplementary works (translation with transcultural approach) before it can be used more widely. International comparative studies should be undertaken on what remains currently an experimental field.

## Additional file

Additional file 1: Characteristics of surveys. (DOC 57 kb)

## Abbreviations

AE: Adverse events
HP: Healthcare professionals
HSOPSC: Hospital survey on patient safety culture
ICPS: International classification for patient safety
KSAB: Knowledge, skills, abilities and behaviours
ME: Medical errors
MS: Medical students
PS: Patient safety
PSC: Patient safety culture
PSI: Patient safety incident
PSMS: Patient safety culture of medical students
WHO: World health organization
