# Supplementary material for: Assessment of patient safety culture: what tools for medical students?
Source: BMC Med Educ. 2016 Sep 29;16:255. doi: 10.1186/s12909-016-0778-y (PMC5043596; doi:10.1186/s12909-016-0778-y)
Supplement: Additional file 1: — Characteristics of surveys. (DOC 57 kb) [file 12909_2016_778_MOESM1_ESM.doc]

| **Additional file 1: Characteristics of surveys** | | | | | | |
| --- | --- | --- | --- | --- | --- | --- |
| **Main author** | **Year**  **-**  **Country** | **Work  achievement context** | **Student (n) (%)** | **Items (n)** | **Parts explored (number of items) and answer modalities**  ***(Y/N: Yes/No – Lx: Likert scale at x modalities – CQM: Multiple Choices Questions – OQ: Open Question)*** | **Statistical validation** |
| **Bechtold LM** | 2007  US | Assess the impact of an educational program on PSMS | 111  (52%) | 20 | 20 items: attitudes related to the shame culture, reporting of adverse events, disclosing medical errors to patients, and training in PS. **(L5)** | - |
| **Carruthers** | 2009  GB | Design a specific tool for assessing PSMS | Survey 1: 364 (30%)  Survey 2: 214 (13%) | 26 | 3 items: training received on PS **(L7)**  3 items: confidence in adverse event reporting system **(L7)**  3 items: influence of workload on error **(L7)** 3 items: medical errors occurring in care **(L7)**  4 items: lack of competence as source of error **(L7)**  3 items: value of medical errors reports **(L7)**  2 items: value of teamwork **(L7)**  2 items: patients' role in preventing errors **(L7)**  3 items: importance of training on PS **(L7)** | CronbachFA et I2C |
| **Coyle YM** | 2005  US | Assess the impact of an educational program on PSMS | 30  (73,3%) | 14 | 5 items: attitudes and behaviour related to incident reporting **(Y/N)**  9 items: perceived barriers to reporting PSIs **(CQM)** | - |
| **Dudas RA** | 2011  US | Assess the impact of an educational program on PSMS | 108  (100%) | 15 | 15 items: attitudes towards patient safety educational programs **(L5)** | - |
| **Flin R** | 2009  GB | Assess the PSMS without specific intervention | 296  (100%) | 33 | 7 items: knowledge of PS **(L5)** 5 items: knowledge of patient safety within clerkship **(L5)**  8 items: opinions after an error **(L5)**  6 items: attitudes towards PS **(L5)**  7 items: behaviour of caregivers (attitudes) **(L5)** | Cronbach |
| **Friedman SM** | 2010  Canada | Assess the PSMS without specific intervention | 220  (37,3%) | 23 | 2 items: care procedures performed without feeling competent **(OQ)** 1 item: reporting care procedures performed without feeling competent **(L5)**  2 items: perceived barriers to reporting **(OQ)** 3 items: PSIs related to the practice **(Y/N, CQM, OQ)** 3 items: reporting of PSIs related to practice **(CQM)**  12 items: perceived barriers to reporting PSIs **(CQM)** | - |
| **Kaldjian LC** | 2007  US | Assess the PSMS without specific intervention | 400  (75%) | 23 | 1 item: link between the announcement of PSIs and the risk of litigation (CQM)  3 items: planning to announce a hypothetical error (L5)  4 items: experiences of announcements (Y / N)  15 items: attitudes to the announcement of an error (L5) | - |
| **Kerfoot BP** | 2007  US | Assess the PSMS without specific intervention | 640  (92%) | 15 | 1 items: self-assessment of knowledge of PS **(L5)**  14 items: skills in PS **(CQM)** | CronbachTest-retest |
| **Leung GK** | 2010  China | Assess the impact of an educational program on PSMS | 130  (83,3%) | 23 | 15 items: attitudes towards medical errors **(L5)**  5 items: self assessment of knowledge about PS **(L5)**  3 items: standpoint on patient safety educational programs **(L5)** | - |
| **Leung GK** | 2010  China | Assess the PSMS without specific intervention | 140  (74%) | 25 | 11 items: attitudes towards PS **(L5)** 6 items: knowledge of PS **(L5)** 8 items: standpoint on patient safety educational programs **(L5)** | - |
| **Logio LS** | 2010  India | Assess the PSMS without specific intervention | 992  (S1 45 S2 53%) | 8 | Study 1: 3 items - reporting experiences of PSI / knowledge of procedures **(Y/N)**  Study 2: 5 items - general experience in formal or informal reporting of PSIs **(L5)** | - |
| **Madigosky WS** | 2006  US | Assess the impact of an educational program on PSMS | 92  (55%) | 31 | 15 items: attitudes towards PS **(L5)**  5 items: skills in PS **(L5)**  5 items: knowledge of PS **(OQ)**  6 items: behaviour **(Y/N)** | - |
| **Moskowitz** | 2007  US | Assess the impact of an educational program on PSMS | 229  (54%) | 23 | 2 items: description of a medical error observed (OQ)  21 items: Attitudes Towards PS (L5) | - |
| **Muller D** | 2007  US | Assess the PSMS without specific intervention | 423  (87%) | 23 | 8 items: definition of medical error (L5)  5 items: experiences of medical errors (Y / N)  2 items: intention of announcing an error (CQM)  1 item: changing perceptions of the error over time? (OQ)  7 items: attitudes to medical errors (L5) | Cronbach |
| **Patey R** | 2007  US | Assess the impact of an educational program on PSMS | Pre-test 70 (64%)  Post-test 38 (29%) | 48 | 7 items: knowledge of PS **(L5)**  6 items: knowledge of actions to be taken after an error **(L5)**  8 items: opinions after an error **(L5)**  4 items: attitudes towards PS **(L5)**  9 items: patient safety during clerkship **(L5)**  7 items: skills in PS **(L5)**  7 items: intentions to behave in the field of PS **(L5)** | - |
| **Sorokin R** | 2005  US | Assess the PSMS without specific intervention | 785  (41%) | 19 | 18 items: attitudes towards PS **(L5)**  1 item: number of PSIs observed / detected **(CQM)** | - |
| **Wetzel AP** | 2011  US | Assess the PSMS without specific intervention | 144  (96%) | 43 | 26 items of Carruthers's survey **(L5)** 13 items: error reporting (or near-miss) **(Y/N)** 4 items: report of a risk behaviour **(L5)** | CronbachFA et I2C |
| **White AA** | 2011  US | Assess the PSMS without specific intervention | 999  (76%) | 11 | 6 items: general attitudes after an error **(CQM)** 5 items: assessment of information that students would give patients **(CQM)** | - |
| PSMS: Patient Safety Culture of Medical Student – PS: Patient Safety - PSI: Patient Safety Incident - FA: Factorial Analysis – I2C: Inter Item Correlation – US: United-States – GB: Great-Britain | | | | | | |
